# White Button Mushroom (*Agaricus bisporus)* Supplementation Ameliorates Spatial Memory Deficits and Plaque Formation in an Amyloid Precursor Protein Mouse Model of Alzheimer’s Disease

**DOI:** 10.3390/brainsci12101364

**Published:** 2022-10-08

**Authors:** Thalia T. Dimopoulos, Stephen L. P. Lippi, Jorge Fernandez Davila, Rachel E. Barkey, Erin N. Doherty, Jane M. Flinn

**Affiliations:** 1 Department of Psychology, Cognitive/Behavioral Neuroscience, George Mason University, Fairfax, VA 22030, USA; 2 Department of Psychiatry and Behavioral Sciences, University of Texas Health Science Center, San Antonio, TX 78229, USA; 3 Department of Biology, George Mason University, Fairfax, VA 22030, USA

**Keywords:** mushrooms, Alzheimer’s disease, spatial memory, amyloid plaque, amyloid, mouse models

## Abstract

Alzheimer’s Disease (AD) is characterized by cognitive impairment and the presence of amyloid-β (Aβ) plaques and tau tangles. This study was conducted to assess the effects of white button mushroom (WBM) supplementation on spatial memory and plaque formation in mice with mutations in amyloid (Aβ). Mice with amyloid precursor protein (hAPP) mutations and their wildtype (WT) littermates were fed a 10% white button mushroom (WBM) feed *ad libitum* three times per week, in addition to their normal diet. Morris water maze (MWM) was conducted at 14 and 32 weeks of age to assess spatial memory and Aβ plaque pathology in the hippocampus was analyzed. Our results showed that hAPP mice on the WBM diet were faster in reaching the platform in the MWM compared to hAPP mice on the control diet at 32 weeks (*p* < 0.05). Significantly fewer plaque deposits were found in the hippocampi of hAPP mice on the WBM diet compared to those on the control diet at 32 weeks (*p* < 0.05). Overall, hAPP mice on the WBM diet had improved spatial memory at 32 weeks of age compared to those on the control diet and exhibited fewer amyloid plaques.

## 1. Introduction

Alzheimer’s Disease (AD) is the sixth leading cause of death in the United States [1] and currently affects 6.2 million Americans [2]. The first symptoms of AD include deficits in spatial memory and spatial awareness [3], where patients become confused while driving, lost on common routes, and have difficulty navigating familiar spaces [4]. One of the main theories for the causation of AD is the formation of amyloid-β (Aβ) plaques, formed by the improper cleavage of the transmembrane protein, the amyloid-beta precursor protein (APP). The proteolytic enzymes β-secretase and γ-secretase cleave APP into the protein Aβ, which accumulates into plaques in areas surrounding neurons [5].

White button mushrooms (WBMs) contain 9.3 micrograms (μg) (Table 1) of selenium in a 100 g/3 oz. serving [6], constituting 16.9% of the recommended daily value (DV) of selenium intake per day for both women and men [7]. Selenium in WBMs is considered an antioxidant [8] that may reduce ROS. As a rich source of selenium, WBMs may be incorporated into a diet-driven approach to reduce AD risk and/or slow symptoms. Other trace minerals may contribute to WBMs’ nutrient density, including potassium, phosphorus, and vitamins B_1_, B_2_, B_12_, C, D, and E [9]. For centuries, mushrooms have been used as a source of homeopathy for oxidative stress-related illnesses. WBMs grow readily in five of the world’s continents, making it one of the most readily available edible fungal varieties which thrive in most agricultural conditions [9]. Beneficial effects of *Agaricus bisporus (A. bisporus)*, the world’s most consumed mushroom, have been seen in metabolic syndrome, immune function, gastrointestinal health, and cancer; with the strongest evidence for the improvement in Vitamin D status in humans [10]. Roncero-Ramos and Delgado-Andrade [11] concluded that the consumption of mushrooms as a part of daily diet could be a natural adjuvant for the treatment and prevention of several chronic diseases. Other studies in mice also showed that powdered *Ganoderma lucidum* (Reishi mushrooms) improved learning and memory, and mimicked nerve growth factor (NGF) activity [12].

In a study conducted by Bennett et al. [13], APPswe/PS1dE9 transgenic mice were administered 5% (wt/wt) WBMs fortified with Vitamin D2 (VDMs) *ad libitum* for 28 weeks. At 36 weeks of age, WT mice on the VDM diet had improved spatial memory, which was measured using the Morris Water Maze (MWM), compared to WT mice not on the diet. There were no dietary effects in AD mice. However, AD mice on the VDM diet exhibited fewer Aβ42 plaques, and had increased levels of APP’s non-amyloidogenic products [13].

In a community cross-sectional study in Singapore, human participants over the age of 60, who self-reportedly consumed more than two portions of various varieties of mushrooms per week, had a 43% reduced risk of acquiring mild cognitive impairment (MCI). Additionally, mushrooms were shown to correlate with faster performance during cognitive examinations in those aged 60 and older [14].

In the present study, a 10% WBM diet was administered three times a week to human amyloid precursor protein (hAPP) mice and WT mice to assess its cognitive benefits. Morris Water Maze (MWM) was conducted at 14 and 32 weeks of age to mirror the timelines before and after Aβ plaques became histologically visible. Congo red histological staining was used to assess quantitative differences in Aβ plaques. This is the first study administering quantity controlled WBMs, without modulations in mineral and vitamin content, to a mouse model of AD.

The purpose of this study was thus to determine whether a diet fortified with wet food containing 10% WBM over the course of six-and-a-half months (29 weeks) would ameliorate the plaque pathology and cognitive deficits of Aβ overexpression in an hAPP mouse model. The first MWM trials occurred at 14 weeks at a time when Aβ plaques originally form at approximately 12 weeks [15]. The time for the second MWM trials at 32 week was chosen to lie between the original period specified by Mucke et al. [16], where robust plaque loads begin at approximately 20–28 weeks of age, and that of Bennett et al. of 36 weeks MWM [13].

## 2. Materials and Methods

### 2.1. Breeding

Offspring were produced from a cross between wildtype C57BL/6J (B6; WT) (Jackson Laboratories, Bar Harbor, ME, USA) female mice and male J20/hAPP (Swe/Ind) mice. Offspring were genotyped by Transnetyx, Inc. (Cordova, TN, USA). Those kept for analysis included mice genotyped as hAPP, and littermates without the hAPP gene (WT mice). Offspring were weaned at 21–28 days.

### 2.2. Housing

Mice were kept in separate, rat-sized cages (Animal Care Systems; Centennial, CO, USA) and assigned to cages based on sex, diet, and genotype. They were placed in ventilated cage racks, with no more than four mice housed in an individual cage. All cages contained TekFresh unautoclaved bedding (Envigo; Indianapolis, IN, USA) and enrichment, which included a running wheel, igloo, and a Nyla-bone (Bio-Serv; Raritan Township, NJ, USA). Mice had access to lixit water *ad libitum* sourced from the cages’ water supply, as well as the WBM or control diet in addition to *ad libitum* unautoclaved Teklad 7012 feed (Teklad 7012, Envigo). Animal care husbandry included daily checks. This study was carried out in accordance with the National Institute of Health’s guide for the care and use of laboratory animals and was approved by George Mason University’s Institutional Animal Care and Use Committee.

### 2.3. Groups

There were four groups in this study, namely hAPP mice on the WBM diet, WT mice on the WBM diet, hAPP mice on the control diet, and WT mice on the control diet (Table 2). Mice surviving both the 14- and 32-week behavioral trials were included in final statistical analyses, as a 30.2% mortality occurred in the total hAPP population across both dietary conditions (*n* = 13). This was potentially due to the hyperactivity of mice with the hAPP transgene, which has been shown in previous studies to be associated with seizures and in some cases fatality [17]. It has been reported by Jackson Laboratories (Bar Harbor, ME, USA) that hAPP (Swe/Ind) mice have a 20% mortality of 12–16 weeks of age, and in other studies, a range of 20–40% mortality from birth to 32 weeks of age was observed [18].

### 2.4. Diet

Mice were administered Teklad 7012 (Envigo) *ad libitum* throughout the duration of this study, from 3 weeks of age until 32 weeks. Giorgio Fresh™ WBMs (Giorgio Foods, LLC; Blandon, PA, USA) were kept frozen in a −80 °C freezer for up to one month at a time; then, they were mechanically blended into the Teklad 7012 feed at a 10% weight-by-volume (*w*/*v*). The dry food was ground up into a powder and water was added to form a paste. Water used to hydrate both the WBM, and control feeds was sourced from lixits attached to the housing racks, as wet food is more palatable to mice than in a powdered suspension. WBM pilei (caps) and lamellae (gills) were incorporated into the WBM feed while the stipes (stems) were discarded, as they contain little to no nutritional value [6]. Each mouse was administered 20.0 (±0.4) grams of either 10% WBM feed or control wet food on feeding days. This was conducted to ensure adequate consumption of the WBMs by providing them in a feed the mice were accustomed to eating.

The amount of WBMs in the mice’s fortified food was increased to 10% compared to the study of Bennett et al. [13], in which a 5% (*w*/*w*) WBM diet was used. The mice on the control diet were fed wet food, without WBM supplementation, three times a week along with their dry food pellets. The mice started the 10% WBM diet at 3 weeks of age and consumed the experimental diet three times per week throughout the duration of the experiment; both groups also received dry food pellets. The mice ate *ad libitum*, ensuring the experimental mice consumed adequate quantities of WBMs. The 10% WBM wet food feedings were weighed prior to being administered and 24 (±4) hours after wet food dispensation to ensure consumption. Additionally, dry food pellets were weighed to determine if the full amount of *ad libitum* consumption of the daily feed was different in the presence of the WBM or control wet diet.

Body weights were collected every two weeks. Individual consumption values were obtained when mice were individually housed for 8 days during circadian activity assessments. No significant effects were noted, and the results are not reported here.

### 2.5. Morris Water Maze

The Morris Water maze (MWM) (*n* = 48, Table 2) is a test of spatial memory where a mouse must utilize visual cues to locate a hidden platform in a pool of water [19]. Spatial cues were placed around the pool, with one outside each quadrant of the pool. Each mouse was placed in the pool facing the wall at a particular cue, in which the starting cue changed daily. When each trial began, the mouse was placed at a designated cue and activity was recorded via the TopScan system (CleverSys, Inc.; Reston, VA, USA). Three 60 s trials occurred for each mouse, and between each trial, a 45 s intertrial interval was provided as previously described [20,21,22]. The MWM paradigm occurred over the course of eight days: the first six days were acquisition trials, the seventh day was a single, 24 h probe trial, and the eighth day used a visual platform to exclude mice with motor deficits. The primary variables of interest in the MWM task were latency to platform (the average amount of time (s)) taken by each animal to locate the escape platform), number of platform crossings on probe trials, and thigmotaxicity. Thigmotaxicity, a measure of anxiety [23,24] is the percentage of time spent swimming in the outer 10% of the pool.

### 2.6. Congo Red Staining

Whole, flash-frozen mouse brains were sliced coronally in a −20 °C cryostat (Leica, CM1510-3) at 16 µm (*n* = 12; Table 3). Each brain was sliced at the anterior, medial, and posterior portions of the hippocampus at approximately 1.31 mm to −2.53 mm from bregma, as found in the Franklin and Paxinos atlas [25]. Six slices were collected per region of the hippocampus, yielding 18 total slices per post-mortem brain. A Congo red amyloid stain kit was used for Aβ plaque detection (Sigma Aldrich, HT-60-1KT). First, slides (Fisher Scientific, Superfrost Plus) were placed into Meyer’s Hematoxylin and incubated for 10 min; then, they were rinsed under running tap water for 5 min. The slides were placed in an alkaline sodium chloride solution for 20 min, then left to incubate in the Congo Red solution for 30 min. Afterwards, the slides were rinsed in three changes of 100% EtOH, dipped in xylene, coverslipped with DPX mountant (Sigma Aldrich, 06522), and left to dry overnight. Slides were subsequently imaged using a brightfield microscope (Olympus, BX-51) with a violet-pigmented lens placed over the lightbulb beneath the microscope’s stage. Microscope settings were 40× magnification with a 1/600 exposure and 200× magnification with a 1/28 exposure. Plaques were subsequently and manually counted by scorers blinded to the experimental condition, as shown in previous literature [26]. Plaques localized in the CA1, CA3, and dentate gyrus regions of the hippocampus were counted.

### 2.7. Statistical Analyses

Statistical analyses were performed with SPSS v. 20.0 (IBM SPSS Statistics for Windows, Armonk, NY, USA) and GraphPad Prism version 8.2.0 (GraphPad Software, San Diego, CA, USA). Graphs were created using GraphPad Prism version 8.2.0 for Mac (GraphPad Software, San Diego, CA, USA). *p* < 0.05 was considered significant and Bonferroni post-hoc analyses followed any significant main effects. All error bars represent the standard error of the mean (SEM).

In MWM analyses, when Mauchly’s test of sphericity was violated, Greenhouse–Geisser corrections were used. Latency to find the platform was analyzed using a 2 (genotype conditions) × 2 (dietary conditions) × 6 (acquisition days of the MWM paradigm) mixed ANOVA. For platform crossings and thigmotaxicity, 2 (genotype conditions) × 2 (dietary conditions) × 3 (Atlantis probe trials) mixed ANOVAs were performed. The single probe trial on day 7 used a 2 (genotype conditions) × 2 (dietary conditions) factorial ANOVA.

In Congo Red plaque counting, two raters blind to the dietary conditions counted the number of plaques and intra-class correlations were calculated to ensure reliability between the raters. The cumulative number of plaques found in the early, medial, and late coronal hippocampi sections of 32-week-old hAPP tissue were quantified. Average plaque values from all regions of each hippocampus were analyzed using a one-tailed *t*-test.

## 3. Results

### 3.1. Feeding

Individually housed mice of all conditions, on average, consumed 86.6% of their wet food at 3 months of age and 86.5% at 8 months. This was equal to approximately 17.3 g of the total 20 g wet food available per mouse. Each mouse was also administered 150 g of Teklad 7012 food pellets *ad libitum*. Mice of all conditions consumed 2% of their dry food pellets at 3 months of age and 3% at 8 months. This was approximately 3.8 g of food pellets over the course of 8 days. No significant differences were observed in the consumption of the dry food pellets when the wet diets were presented. There were no significant differences in body weight between diet groups.

### 3.2. Morris Water Maze at 14 Weeks

**Figure 1:**
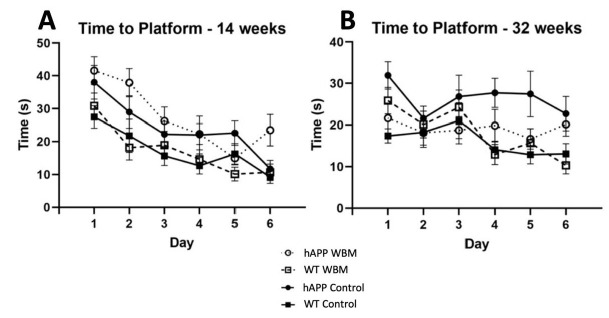
Morris Water Maze Performance (*n* = 48). (**A**) For latency to find the platform in MWM at 14 weeks, there was a significant effect of day where mice found the platform more quickly as days progressed (*p* < 0.001). (**B**) For latency to find the platform in MWM at 32 weeks, there was a significant effect of day, where mice of both genotypes found the platform faster as the days progressed (*p* < 0.01). In addition, hAPP mice on the WBM diet had a shorter latency than hAPP mice on the control diet (*p* < 0.05).

There was a significant effect across days, where mice were quicker to the location of a hidden platform as they progressed (*F*_4.186,184.176_ = 19.907, *p* < 0.001, partial η^2^ = 0.312) (Figure 1A). A significant genotype effect was evident at 14 weeks for latency (*F*_1,44_= 20.083, *p* < 0.001, partial η^2^ = 0.313). hAPP mice had a significantly higher latency compared to WT mice. There was no significant diet effect in latency to find the platform (*p* > 0.05). There was a significant effect of day across probe trials, where thigmotaxicity decreased as testing days progressed (*F*_1.595,70.161_ = 10.799, *p* < 0.001, partial η^2^ = 0.197). There was a significant effect of diet (*F*_1,44_ = 4.748, *p* = 0.035, partial η^2^ = 0.097) where hAPP mice on the WBM diet had higher levels of thigmotaxicity than the control diet in the three probe trials. hAPP mice had significantly higher levels of thigmotaxicity compared to WT mice (*F*_1,44_ = 11.866, *p* = 0.001, partial η^2^ = 0.212) across the three probe trials. During the seventh day 24 h probe trial, there was also a significant effect of diet (*F*_1,44_ = 10.025, *p* = 0.003, partial η^2^ = 0.186).

There was a significant effect of day for increased crossings over the platform on probe days 2, 4, and 6 (*F*_2,88_ = 7.334, *p* = 0.001, partial η^2^ = 0.143). No significant interaction between diet and genotype was noted. However, a significant genotype effect was noted (*F*_1,44_ = 14.482, *p* < 0.001, partial η^2^ = 0.248), where hAPP mice made significantly fewer crosses over the platform than WT mice across probe trials for the first three probe days. On the seventh day 24-h probe trial, this genotype effect was also significant where hAPP mice made fewer crosses over than WT mice (*F*_1,44_ = 5.358, *p* < 0.05; partial η^2^ = 0.109). No diet effect was seen in crossings throughout the probe trials.

### 3.3. Morris Water Maze at 32 Weeks

There was an effect of day for latency to find the platform (*F*_5,220_ = 3.440, *p* < 0.01, partial η^2^ = 0.073) (Figure 1B). A significant effect of genotype was seen (*F*_1,44_ = 10.785, *p* = 0.002, partial η^2^ = 0.197), where hAPP mice took significantly longer to find the platform than WT mice. There was no significant effect of diet on latency at 32 weeks of age. However, a significant diet by genotype interaction (*F*_1,44_ = 7.245, *p* = 0.01, partial η^2^ = 0.141) was noted. A simple effects analysis showed that hAPP mice on the control diet took significantly longer to find the platform than hAPP mice receiving the WBM diet (*p* < 0.01), signifying mediated latency in WBM-supplemented hAPP mice. The hAPP mice on the control diet also took significantly longer to find the platform compared to WT mice on the control diet (*p* < 0.001) (See Figure 1B).

Thigmotaxicity decreased across probe trials (*F*_2,88_ = 5.762, *p* = 0.004, partial η^2^ = 0.116) and there was a significant day by genotype interaction (*F*_2,88_ = 4.186, *p* = 0.018, partial η^2^ = 0.087). A simple effects analysis revealed that on day 4, control hAPP mice spent significantly more time in the outer area than hAPP WBM mice (*p* = 0.034). While hAPP mice did not improve significantly over time, WT mice did, showing less anxiety on days 4 (*p* < 0.001) and 6 (*p* = 0.001) compared to day 2. There was no significant effect of diet on thigmotaxicity at 32 weeks of age.

A significant between-subjects effect of genotype was seen for the number of crosses across the probe trials (*F*_1,44_ = 7.189, *p* = 0.01, partial η^2^ = 0.140). hAPP mice made significantly fewer crossings over probe days compared to WT mice. There was no significance for the seventh day probe trial for crosses at 32 weeks. There was no significant diet effect for crosses at 32 weeks of age.

### 3.4. Congo Red Staining

**Figure 2:**
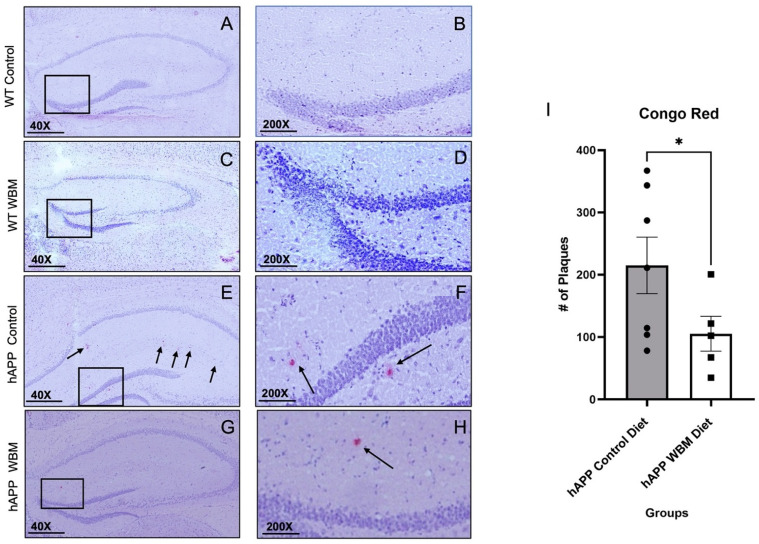
Congo red staining in the Hippocampus (*n* = 12). (**A**–**D**) No plaques were found in WT mice, as expected. Subfiigures (**B**,**D**,**F**,**H**) are expanded versions of the boxes in subfigures (**A**,**C**,**E**,**G**); the arrows indicate specific plaques within the medial hippocampal area. (**E**,**F**) Congo Red staining in an hAPP control diet mouse. (**G**,**H**) Congo Red staining in an hAPP mouse on the WBM diet. (**I**) hAPP mice on the WBM diet had significantly fewer plaques than those on the control diet (*p* < 0.05). (* *p* < 0.05). Scale bar at 40× magnification is 500 μm and at 200× magnification the scale bar is set to 100 μm.

There was strong agreement between the two raters’ plaque counts: ICC = 0.984, *p* = 0.001. The two raters’ counts were thus averaged for analysis. No Aβ plaques were found in WT mice of either dietary condition, as anticipated (Figure 2A–D). Averaging the Aβ plaques found per brain showed that hAPP mice on the WBM diet had significantly fewer Aβ plaques compared to hAPP mice on the control diet (*t*_10_ = 1.86, *p* < 0.05) (Figure 2E–I).

## 4. Discussion and Conclusions

The beneficial effects of WBM administration in this hAPP mouse model are evident in the improved performance in the MWM testing and reduced Congo red histology seen at 32 weeks of age. The WBM diet appeared to ameliorate the spatial memory deficits seen in transgenic AD mice as they increase in age [27].

Analysis of spatial memory through the MWM showed that 14-week-old hAPP mice took significantly longer than WT mice in both dietary conditions to find the platform. At this age, hAPP mice showed greater thigmotaxicity than WT mice regardless of diet, but mice on the WBM diet displayed greater anxiety than control diet mice. Therefore, the WBM diet seemed to be ineffective at 14 weeks regarding latency, thigmotaxicity, or crossings. However, at the 32-week time-point in later disease progression, hAPP mice on the WBM diet found the platform significantly faster than hAPP mice on the control diet. hAPP mice fed the WBM diet also showed decreased anxiety, which is normally seen in hAPP mice [28], as shown by the reduced thigmotaxicity within the maze. In a recent study, an AD tau mouse model exhibited anxiolytic effects following Lion’s mane (*Hericium erinaceus*) mushroom administration over the course of four months [29]. Additionally, this indicates that WBM supplementation could be ameliorative when administered for longer periods of time. Potentially, WBMs may be incorporated therapeutically in patients with the present symptoms of cognitive impairment, as mushrooms have already been shown to reduce risk of mild cognitive impairments in older individuals [14]. Future studies could assess anxiety through Elevated Zero Maze (EZM) and Open Field Testing (OFT). These effects of WBMs may be due to several factors, including selenium. Future studies should directly analyze and assess the effects of selenium on learning and memory.

Although these spatial memory findings show that WBMs may be a potential therapeutic agent, other studies, such as that of Bennett et al. [13], were unable to find significance in spatial memory in transgenic AD mouse models while on a WBM diet. In the present study, the WBM diet was administered three times per week versus daily compared to the study of Bennett et al. [13]. The percentage of the WBM regimen in the current study was increased to 10% three times per week in a wet food form, versus 5% daily in a pellet form [13]. The differences seen in the study of Bennett et al. [13] may be due to the lower dosing regimen compared to the current study or the use of a different mouse model, the APP_Swe_/PS1dE9 mouse. Normally, hAPP mice are not expected to perform at the same level as WT mice; however, at 32 weeks of age, as shown in Figure 1B, the hAPP mice on the WBM diet did not differ significantly from WT controls.

Feng et al. [14] showed that in humans, at least three weekly servings of WBMs mitigated MCI in elderly patients. Our study shows these benefits in mice when consumption begins at an early age (4 weeks) throughout middle age (32 weeks). Considering that the hAPP phenotype is associated with an early and familial onset of AD, the 32-week old mice used in the study mirror the onset of symptoms in humans [27]. As recommended by the FDA (Table 1) [6], a 3 oz. serving is considered as the appropriate dose of WBMs for human consumption. A portion was considered in the work of Feng et al. [14] to be a ¾ cup of mushrooms, of approximately 150 g, where a dose of greater than 300 g weekly reduced MCI scores.

Congo red staining showed the hAPP mice on the WBM diet exhibited fewer plaques in their hippocampi, reinforcing WBM’s capacity to ameliorate plaque deposition. These histological findings further reinforce the capability of WBMs to improve both physiological and neuropsychological deficits discovered in this hAPP mouse. This reduction in Aβ plaque loads in the hippocampal tissue could be due to WBM’s antioxidant effect, which has been shown to reduce Aβ accumulation [12]. Future studies could explore the effects of WBMs on other brain markers, including inflammation and soluble amyloid.

This study has shown the benefits of WBM dietary intake in an hAPP AD mouse model. Since this is an accessible food for most individuals, it shows great translatability for humans. This study was conducted to bring attention to the importance of environmental factors in mitigating neurodegenerative diseases, such as AD. WBMs are the most readily available mushroom in the US [30] and are accessible nationally. The WBMs grown by Giorgio Fresh (Giorgio Foods, LLC) are sold in superstores nationwide; most of these accept food stamps, making WBMs affordable and accessible to people from all economic backgrounds.

## Figures and Tables

**Table 1:** White Button Mushroom Concentrations Sample (USDA, 2018).

Nutrient	Amount in a 100 Gram (g) per 3 Ounce (oz) Serving	Unit
Protein	3.09	g
Carbohydrates	3.26	g
Potassium	318	Milligrams (mg)
Iron	0.5	mg
Selenium	9.3	Micrograms (μg)
Zinc	0.52	mg
Copper	0.32	mg
Sodium	5.0	mg

**Table 2:** Number of Mice used in MWM.

	hAPP (21)	WT (27)
**WBM**	11 (4F, 7M)	14 (7F, 7M)
**Control**	10 (6F, 4M)	13 (5F, 8M)

**Table 3:** Number of Mice used in Congo Red.

	hAPP (12)
**WBM**	5 (1F, 4M)
**Control**	7 (3F, 4M)

## Data Availability

The data presented in this study are available upon request from the corresponding author, without undue reservation.
